# Understanding the dynamics of Toll-like Receptor 5 response to flagellin and its regulation by estradiol

**DOI:** 10.1038/srep40981

**Published:** 2017-01-23

**Authors:** Ignacio Caballero, James Boyd, Carmen Almiñana, Javier A. Sánchez-López, Shaghayegh Basatvat, Mehrnaz Montazeri, Nasim Maslehat Lay, Sarah Elliott, David G. Spiller, Michael R. H. White, Alireza Fazeli

**Affiliations:** 1 Academic Unit of Reproductive and Developmental Medicine, Department of Oncology and Metabolism, The Medical School, University of Sheffield, Sheffield, United Kingdom; 2 ISP, INRA, Université Tours, 37380, Nouzilly, France; 3 Michael Smith Building, Faculty of Life Sciences, University of Manchester, Manchester, United Kingdom; 4 Institute of Biomedicine and Translational Medicine, Department of Pathophysiology, University of Tartu, Tartu, Estonia

## Abstract

Toll-like receptors (TLRs) are major players of the innate immune system. Once activated, they trigger a signalling cascade that leads to NF-κB translocation from the cytoplasm to the nucleus. Single cell analysis shows that NF-κB signalling dynamics are a critical determinant of transcriptional regulation. Moreover, the outcome of innate immune response is also affected by the cross-talk between TLRs and estrogen signalling. Here, we characterized the dynamics of TLR5 signalling, responsible for the recognition of flagellated bacteria, and those changes induced by estradiol in its signalling at the single cell level. TLR5 activation in MCF7 cells induced a single and sustained NF-κB translocation into the nucleus that resulted in high NF-κB transcription activity. The overall magnitude of NF-κB transcription activity was not influenced by the duration of the stimulus. No significant changes are observed in the dynamics of NF-κB translocation to the nucleus when MCF7 cells are incubated with estradiol. However, estradiol significantly decreased NF-κB transcriptional activity while increasing TLR5-mediated AP-1 transcription. The effect of estradiol on transcriptional activity was dependent on the estrogen receptor activated. This fine tuning seems to occur mainly in the nucleus at the transcription level rather than affecting the translocation of the NF-κB transcription factor.

Toll-like receptors (TLRs) are a family of evolutionary conserved pattern recognition receptors (PRRs) from the innate immune system. They are membrane bound-receptors, with up to 10 members in humans and 12 in mice. Traditionally, they are considered to be a part of the first line of defence against pathogens, their major role being to detect specific microbial motifs, known as pathogen-associated molecular patterns (PAMPs) and to trigger an inflammatory response^1^. Abnormal TLR signalling has been related to a variety of pathologies, including acute and chronic infections, autoimmune diseases, immunodeficiencies and cancer^2^,^3^,^4^.

TLRs downstream signalling involves the activation of mitogen-activated protein kinases (MAPK) and the nuclear factor κB transcription factor (NF-κB)^1^. This transcription factor family is formed by 5 members, RelA, RelB, c-Rel, p50/p105 (NF-κB1), and p52/p100 (NF-κB2). In the absence of stimuli, NF-κB proteins are bound in the cytoplasm to proteins from the inhibitor of nuclear factor κB (IκB) protein family, preventing NF-κB translocation to the nucleus. Upon stimulation, IκBα is phosphorylated by the IκB kinase (IKK) and degraded, allowing the NF-κB dimers to move into the nucleus and bind to the DNA, triggering the expression of target genes^5^,^6^. After translocation into the nucleus, NF-κB target genes including cytokines and negative feedback regulators of the system, such as IκB and A20, that have the potential to produce oscillations^7^. NF-κB is a major network hub processing many different inflammatory signals that lead to different transcriptional response. However, understanding how this genetic circuit can provide the adequate transcriptional response to each stimulus is unclear. Single cell studies and mathematical modeling are clarifying the biological significance of the dynamics of several signalling pathways (especially NF-κB) and their oscillatory behavior^8^,^9^. They show that the type (TNF-α, LPS) and length of the stimulus can produce different patterns of NF-κB oscillatory dynamics, which may code for different transcriptional response and cell fate^5^,^10^,^11^,^12^.

The immune response is also regulated by cross-talk between signalling pathways. Differences in the male and female immune response have been observed, with women having an increased tendency to suffer autoimmune diseases. Hormones are thought to play an important role in these sex-related differences^13^. Estrogens are known to modulate the immune response, producing both pro- and anti-inflammatory effects through the activation of different estrogen receptors (ERs)^14^,^15^. The classical mechanism through which nuclear ERs (ERα and ERβ) are able to regulate transcription involves ER binding to estrogen response elements (ERE) in the promoter region of target genes. In addition, ERs can also modulate gene expression without a direct interaction with the DNA sequence by binding to other transcription factors, such as NF-κB and AP-1. Estrogens can also modulate signal transduction pathways through a non-genomic route that involves the activation of the G protein-coupled receptor GPR30. Activation of GPR30 stimulates Ca^2+^ mobilization, production of cAMP and induction of MAPK signalling in a variety of cells^16^,^17^. This signalling can extend to AP-1 and NF-κB transcription factors, resulting in gene repression or activation^18^.

TLR5 is expressed in lung and intestinal epithelial cells, human endometrium, bladder, granulosa cells leukocytes, adipocytes, and some cancer cells, among others. Its signalling is triggered upon recognition of the flagellin protein component of bacterial flagella. TLR5 plays a prominent role in several physiological and pathological processes, such as embryo implantation, breast cancer, insulin resistance, and maintenance of intestinal and lung homeostasis^19^,^20^,^21^,^22^,^23^,^24^. Modulation of TLR5 functionality by estrogens is still unclear. Cross-talk between ERs and other transcription factors seems to play an important role in the regulation of TLR5 mediated gene expression^25^. Incubation of human bladder epithelial cells with estradiol (E2) downregulates TLR5 expression but increases TLR5-dependent IL-6 secretion^26^. However, no response to flagellin is observed in E2 producing granulosa cells that express TLR5^27^,^28^.

In the present study, we aimed to increase our understanding of TLR5 signalling and its modification by hormones. We measured NF-κB signalling dynamics mediated by TLR5 activation and its modulation by E2 via different ERs (ERα, ERβ and GPR30). In particular, we focused on the determination of TLR5-mediated NF-κB nuclear localization dynamics at the single cell level and NF-κB and AP-1transcription activity in MCF7 cells, a hormone responsive breast cancer cell line^29^.

## Results

### Flagellin causes a single RelA translocation event into the nucleus that is not affected by estradiol

**Figure 1:**
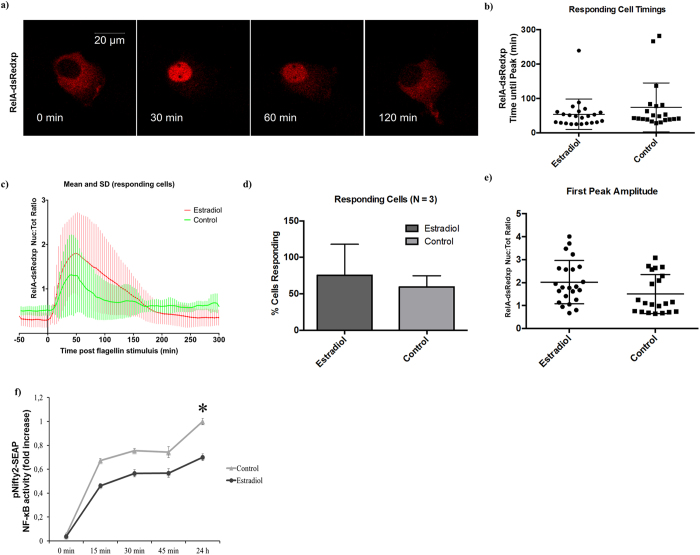
Estradiol does not affect RelA translocation into the nucleus. MCF7 cells were pre-incubated or not (control) with 10 nM E2 for 24 h and stimulated with 100 ng/ml of flagellin. (**a**) Time-lapse confocal images of a typical RelA-dsRedxp translocation after flagellin stimulation. (**b**) Time to nuclear RelA peak in MCF7 cells transiently transfected with RelA-dsRedxp. (**c**) Time course of RelA-dsRedxp normalized amplitude in transiently transfected responding MCF7 cells after flagellin stimulation. (**d**) Percentage of RelA-dsRedxp transiently transfected MCF7 cells responding to flagellin. (**e**) Amplitude and area of RelA-dsRedxp transiently transfected MCF7 cells after flagellin stimulation. (**f**) SEAP expression of MCF7 cells transiently transfected with pNifty2-SEAP incubated with 100 ng/ml of flagellin for 0, 15, 30, 45 min or 24 h. SEAP expression was evaluated 24 h post flagellin stimulation. NF-κB transcription activity was normalized against the control group stimulated for 24 h. Data are representative of 3 independent experiments with more than 20 cells per experiment (A–D) or at least five independent experiments (E). Error bars denote SEM (**d**,**f**) or SD (**b**,**c**,**e**). *P < 0.05.

TLR5 activation by 100 ng/ml of flagellin showed a peak of RelA translocation to the nucleus that ranged from 30 to 100 min post flagellin stimulation, with an average around 60 min. No differences were observed in either amplitude or time to first peak when MCF7 cells where cultured in the presence of 10 nM E2 (Fig. 1a,b). A single NF-κB translocation event was observed in the majority of the cells in response to TLR5 activation using confocal single live-cell fluorescent imaging. No differences were observed when MCF7 cells were cultured in the presence of 10 nM E2 (Fig. 1c and Fig. S2). No differences were observed either in the number of MCF7 cells responding to flagellin or in the amplitude and area under the response curve (which indicates the length of time that RelA stayed in the nucleus), regardless of whether or not they were pre-incubated with E2 (Fig. 1d–e).

To test the effect of the length of flagellin stimulation, MCF7 cells were subjected to short periods of flagellin stimulation (15, 30 or 45 min) and the response in NF-κB transcription activity was compared to samples stimulated for 24 h. We observed that nearly 80% of the total transcription activity could be obtained after just 30 min of flagellin stimulation, regardless of the presence of E2. As expected, a lower NF-κB transcription activity was still observed in the presence of E2 compared to control samples (p < 0.05), which was independent of the period of flagellin stimulation (Fig. 1f). These results showed that a short stimulus with flagellin (15 to 45 min) is able to achieve NF-κB transcription activity levels close to those obtained with long (24 h) stimulus.

**Figure 2:**
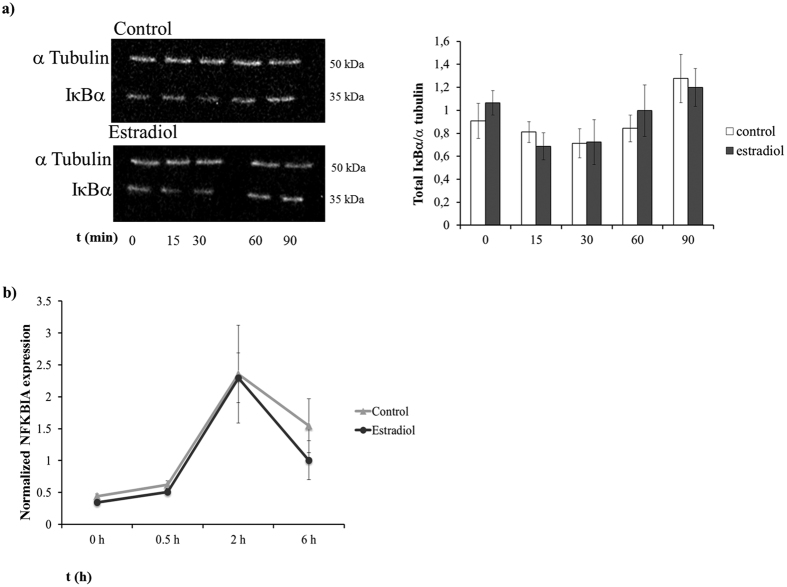
Estradiol does not affect TLR5-mediated IκBα expression. MCF7 cells were pre-incubated or not (control) with 10 nM E2 for 24 h and stimulated with 100 ng/ml of flagellin. (**a**) Western blot of IκBα and α Tubulin of MCF7 cells. (**b**) Real-time qPCR analysis in IκBα gene expression of MCF7 cells cultured in the presence of 10 nM E2 or not (control) and stimulated with 100 ng/ml of flagellin for 0, 0.5, 2 and 6 h before collection. Data are representative of at least 3 independent experiments. Error bars denote SEM.

Western blotting analysis of TLR5-mediated IκBα degradation showed a decrease in IκBα that reached its maximum 30 min after flagellin stimulation. No differences were observed between the control and E2 group (Fig. 2a). Similarly, TLR5 activation triggered the expression of the NFKBIA gene, which peaked at 2 h post-stimulation. No differences were found between the control and the E2 treated cells (Fig. 2b).

### Estradiol decreases TLR5-mediated NF-κB transcription activity

**Figure 3:**
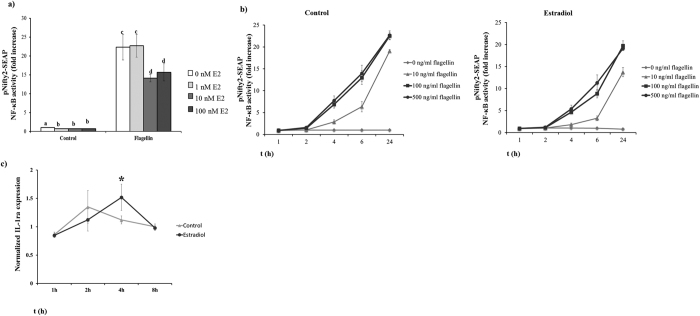
Estradiol decreases TLR5-mediated NF-κB transcription activity. MCF7 cells transfected with the pNifty2-SEAP reporter plasmid were used to evaluate the effect of E2 on NF-κB transcription activity. (**a**) Transfected MCF7 cells were pre-incubated for 24 h with different E2 concentrations (0, 1, 10 and 100 nM) and then stimulated for 24 h with 100 ng/ml of flagellin or not (control group). (**b** and **c**) Transfected MCF7 cells were pre-incubated or not (control) with 10 nM E2 for 24 h. Cells were stimulated with 0, 10, 100 or 500 ng/ml of flagellin and samples collected 1, 2, 4, 6 and 24 h post-stimulation. (**b**) NF-κB transcription activity is reported as accumulated SEAP expression. Samples were analyzed using Quantiblue^TM^. Data of NF-kB activity are reported as the fold induction of SEAP activity over untreated controls. (**c**) Real-time qPCR analysis of IL-1ra gene expression in MCF7 cells cultured in the presence of 10 nM E2 or not (control) and stimulated with 100 ng/ml of flagellin for 1, 2, 4 and 8 h before collection. Data are representative of at least three independent experiments. Error bars denote SEM. Different letters mean significant difference (p < 0.05). *Indicates p < 0.05.

An important feature of estrogen is that its effect on the cells is dose-dependent^15^. When we tested the effect of different concentrations of E2 (0, 1, 10 and 100 nM) on the TLR5-mediated NF-κB activity, we observed that a minimum of 10 nM E2 was needed to decrease NF-κB transcriptional activity (p < 0.05). No differences were found at higher concentrations up to 100 nM (Fig. 3a). Treatment of MCF7 cells with different concentrations of flagellin (10, 100 and 500 ng/ml) significantly increased NF-κB transcriptional activity in a concentration-dependent manner (P < 0.05). No further increase was observed with flagellin concentrations above 100 ng/ml. This increase in NF-κB transcriptional activity was significantly lower when the cells were incubated in the presence of E2 for all flagellin concentrations (Fig. 3b p < 0.05). A more detailed analysis of the dynamics of SEAP secretion showed that the higher amounts of transcription activity occurred between 2 and 6 h post-flagellin stimulation when MCF7 cells where stimulated with higher doses of flagellin (100 and 500 ng/ml) in the absence of E2. However, when MCF7 cells where cultured in the presence of 10 nM E2, the increase in NF-κB transcription activity was delayed, and the peak of NF-κB activity was observed between 4 and 6 h after flagellin stimulation (Fig. 3b). The dynamics of IL-1ra gene expression, an NF-κB-dependent gene^30^, were delayed when cells were cultured in the presence of 10 nM E2. IL-1ra gene expression peaked at 2 h post-flagellin stimulation in control MCF7 cells compared to 4 h in the presence of 10 nM E2 (p < 0.05) (Fig. 3c).

### Estradiol modulates TLR5 gene expression

**Figure 4:**
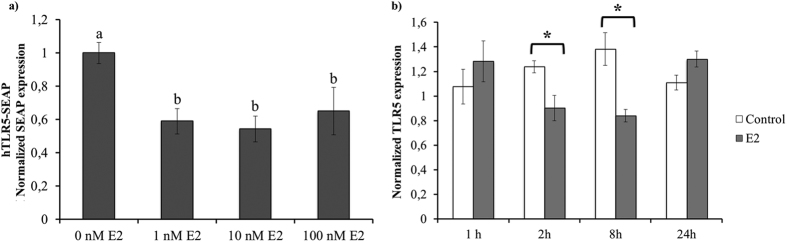
Estradiol modulates TLR5 gene expression. (**a**) Down-regulation of SEAP expression in MCF7 cells transfected with the hTLR5p-SEAP reporter plasmid after 24 h incubation with 1, 10 and 100 nM E2. Samples were analysed using NovaBrightTM Secreted Placental Alkaline Phosphatase (SEAP) Enzyme Reporter Gene Chemiluminescent Detection System 2.0. SEAP expression is reported as the fold induction of treated over untreated controls. (**b**) Real-time qPCR analysis of TLR5 gene expression of MCF7 cells cultured in the presence of 10 nM E2 or not (control) for 1, 2, 8 and 24 h. Data are representative of at least five independent experiments. Error bars denote SEM. Different letters mean significant difference (p < 0.05). *Indicates p < 0.05.

To evaluate whether E2 had a direct effect on TLR5 function by binding to its promoter and affecting gene expression, the upstream regions of the TLR5 gene were examined. Analysis of putative binding sequences identified two ERα binding motifs (−2176 and −1835) (Fig. S1). A TLR5 expression reporter (hTLR5p-SEAP) where the TLR5 promoter (including those ERα motifs) drives the expression of SEAP was generated. E2 inhibited transcription from the TLR5 promoter in MCF7 cells 24 h after flagellin addition regardless of the E2 concentration used (p < 0.05; Fig. 4a). E2 addition to flagellin treated MCF7 cells produced a down regulation in TLR5 expression (as measured by qPCR) from 2 to 8 h post-stimulus (p < 0.05), returning to a similar TLR5 expression after 24 h (Fig. 4b).

### Estradiol increases TLR5-mediated AP-1 activity

**Figure 5:**
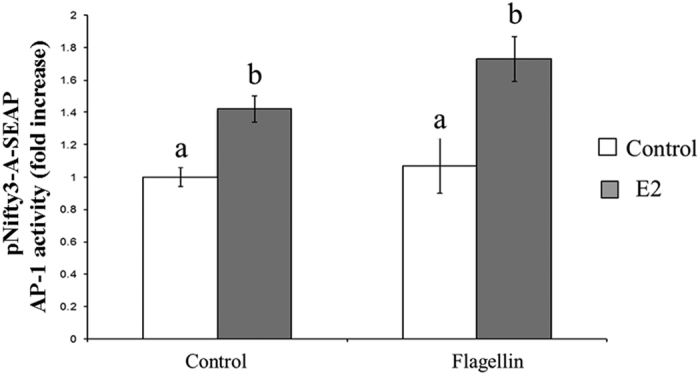
Estradiol increases TLR5-mediated AP-1 transcription activity. MCF7 cells transfected with the pNifty3-A-SEAP reporter plasmid were pre-incubated or not (control) with 10 nM E2 for 24 h and stimulated with 100 ng/ml of flagellin for 24 h. Data of AP-1 activity are reported as the fold induction of SEAP activity over untreated controls. Data are representative of at least five independent experiments. Error bars denote SEM. Different letters mean significant difference (p < 0.05).

As previously described, the canonical TLR5 signalling pathway is able to activate both NF-κB and AP-1 transcription factors. To examine whether E2 modulated AP-1 transcription activity, MCF7 cells were transfected with an AP-1 reporter plasmid (pNifty-3-A-SEAP, Invivogen) and pre-incubated with and without 10 nM E2 for 24 h, before the addition of 100 ng/ml of flagellin. Addition of E2 increased AP-1 activation compared to controls. Furthermore, the addition E2 had a synergistic effect on flagellin-derived AP-1 activation in MCF7 cells (p < 0.05; Fig. 5).

### Differential role of Estrogen receptors in NF-κB and AP-1 activation

**Figure 6:**
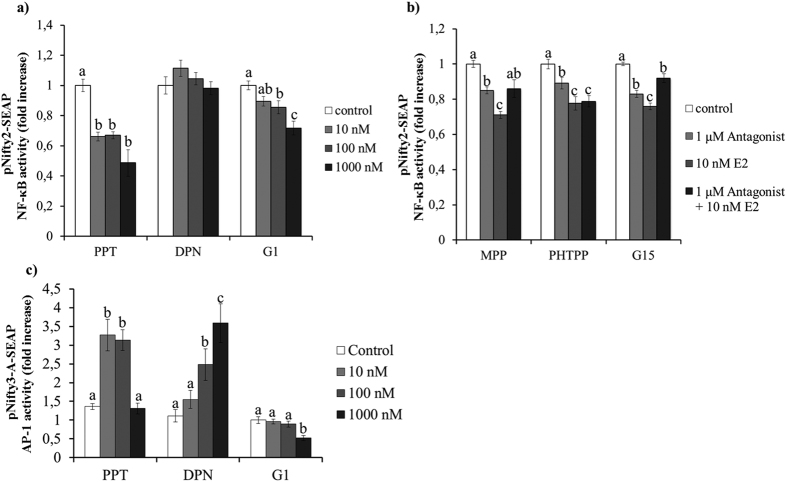
Differential role of Estrogen receptors in NF-κB and AP-1 transcription activity. SEAP expression of MCF7 cells transfected with either the pNifty2-SEAP (**a** and **b**) or the pNifty3-A-SEAP (**c**) reporter plasmid. (**a** and **c**) Transfected MCF7 cells were pre-incubated with ER agonists PPT, DPN or G1 (0, 10, 100 and 1000 nM) for 24 h and then stimulated with 100 ng/ml of flagellin. (**b**) Effect of the ER blockade by specific ER antagonists (MPP, PHTPP or G15). A 2 × 2 factorial experimental design was used where transfected MCF7 cells were either: (1) cultured in the absence of both ER antagonist and E2 (control); (2) treated with 1 μM of ER antagonist for 30 min; (2) pre-incubated with 10 nM E2 for 24 h or (2) treated with 1 μM of ER antagonist for 30 min and then pre-incubated with 10 nM E2 for 24 h. The cells were then stimulated with 100 ng/ml of flagellin and analyzed at 24 h using NovaBrightTM Secreted Placental Alkaline Phosphatase (SEAP) Enzyme Reporter Gene Chemiluminescent Detection System 2.0. Control Data of NF-kB activity are reported as the fold induction of SEAP activity over untreated controls. Data are representative of at least five independent experiments. Error bars denote SEM. Different letters mean significant difference (p < 0.05).

In order to discern the role of these receptors in the observed effect of E2, we tested the effect of different concentrations (10, 100 and 1000 nM) of selective agonists for ERα, ERβ or GPR30 in NF-κB transcription activity. Transiently transfected MCF7 cells were pre-incubated for 24 h with the selective ER agonists and stimulated with 100 ng/ml of flagellin for 24 h. Pre-incubation with ERα agonist propylpyrazoletriol (PPT) and GPR30 agonist (G1) significantly decreased the levels of NF-κB transcription activity (Fig. 6a) (p < 0.05). No effect was observed when MCF7 cells were pre-incubated in the presence of the ERβ agonist diarylpropionitrile (DPN). Then, transfected MCF7 cells were treated with specific antagonists against each of the ERs before pre-incubation for 24 h with 10 nM E2 and subsequent stimulation with 100 ng/ml of flagellin. A 30 min pretreatment of MCF7 cells with either the selective ERα antagonist methylpiperidinopyrazole (MPPdihydrochloride) or the selective GPR30 antagonist G15, prior to incubation with E2, was able to significantly increase NF-κB transcription levels compared to the E2 treated cells (p < 005). No effect was observed when cells were pretreated with the ERβ antagonist pyrazolo [1,5-a] pyrimidin (PHTPP) (Fig. 6b). It is noteworthy that ERα gene expression was significantly downregulated in MCF7 cells in the presence of 10 nM E2 (p < 0.05; Fig. S3).

Interestingly, when MCF7 cells were stimulated with 10 ng/ml of TNF-α instead of flagellin, we observed that both ERα and ERβ, but not GPR30, were able to decrease NF-κB activation (p < 0.05; Fig. S4). Moreover, when we investigated the effect of the different ERs on TLR5 activation of AP-1, we observed that pre-incubation of MCF7 cells with either PPT (ERα agonist) or DPN (ERβ agonist) significantly increased AP-1 transcription activity (p < 0.05). However, no effect was observed when MCF7 cells were pre-incubated with G1 (GPR30 agonist) (Fig. 6c).

## Discussion

Here, we characterized for the first time, the dynamics of TLR5 signalling in response to flagellin by live cell imaging. We observed that most of the cells showed a single translocation with a first peak of RelA translocation around 60 min post-flagellin that was sustained for several hours and led to an increase in NF-κB transcription activity. Although nuclear translocation dynamics were not affected by E2, NF-κB transcription activity was significantly decreased which suggest an effect of the ERs at the nuclear level.

The kinetics of TLR5-mediated RelA nuclear localization observed in our experiments were very similar to those described by Lee *et al*. for TLR4^12^. Stimulation of mouse embryo fibroblasts with LPS, a ligand for TLR4, produced a sustained activation of NF-κB. This sustained NF-κB activation was suggested to be dependent on TRIF activation of a TNF-α paracrine signalling^12^,^31^. The low number of cells displaying secondary and tertiary RelA pulses into the nucleus after TLR5 activation could explain the small differences found in transcription activity when MCF7 incubation time with flagellin was increased from 30 min to 24 h, as well as the low NF-κB activity observed from 6 to 24 h post-stimulation. Our observations are similar to the NF-κB responses that have been described when cells were stimulated with low doses of TNF-α^32^. However, we showed that increasing the flagellin concentration from 100 to 500 ng/ml did not change either the total NF-κB transcription activity nor the dynamics of NF-κB transcription, suggesting that the dose employed (100 ng/ml) had reached saturation. Similar results have been described in different endometrial cell lines, where increasing flagellin concentrations over 100 ng/ml did not increase the production of IL-8^20^. In our experimental system, the addition of 100 ng/ml of flagellin represented approximately 2 × 10^6^ molecules per cell. This number represented about a 10000 fold increase compared to the described number of molecules for low doses of TNF-α^32^. This suggests that the heterogeneity of the response observed after flagellin stimulation is unlikely to come from a stochastic binding of flagellin to TLR5. Instead, it is possible that TLR5-mediated NF-κB activation was dependent on the intrinsic cell status, which would determine whether the TLR5 downstream signalling was activated upon flagellin recognition.

Signalling cascades are not a straightforward process. Intensive cross-talk between signalling pathways play a crucial role in the outcome of the innate immune response. In this regard, it has been known for years that estrogens played an important role in the modulation of inflammation. However, the effect of estrogens on the innate immune system is still unclear. This has been attributed to differences in the inflammatory stimulus and the diversity of ERs available^15^. Understanding this cross-talk may be crucial for the treatment of several inflammatory disorders that are influenced by both TLR5 activation and sex hormones, such as breast cancer, cystic fibrosis and embryo implantation failure^20^,^21^,^33^. In our study, we observed that E2 modulates TLR5-mediated inflammation in a more complex manner than previously thought. Estradiol concentrations higher than 10^−8^ M significantly decreased NF-κB activity *in vitro*. This effect was observed for all flagellin concentrations tested. Other *in vitro* studies using different stimulus, such as TNF-α^34^, Phorbol 12-myristate 13-acetate (PMA)^35^ or LPS^36^,^37^, have shown a decrease in NF-κB activation in those samples incubated in the presence of E2. For example, mouse RAW 264.7 and rat astrocytes showed a decrease in NF-κB transcriptional activity after LPS stimulation^36^,^37^. Along the same lines, NF-κB activation by PMA in HeLa cells was almost completely inhibited by E2 concentrations at levels found during pregnancy^35^.

There are contradictory reports in regard to the mechanisms through which E2 inhibits NF-κB activity. Some reports show that E2 is able to prevent RelA nuclear translocation in macrophages and in a lower proportion in MCF7 cells^37^. On the other hand, Xing *et al*.^38^ did not observe any effect of E2 on RelA translocations and suggested that E2 inhibition of NF-κB was dependent on an increased IκBα production and the blocking of RelA binding sites in the promoter region of pro-inflammatory genes. Our results are in agreement to those presented by Xing *et al*.^38^ since we did not observe any effect of E2 on the dynamics of NF-κB translocation measured at the single cell level in any of the parameters analysed. However, we did not find any changes in IκBα expression at the gene or protein level. It is noteworthy that a different inflammatory stimulus (TNF-α vs flagellin) and method of detection for RelA translocations was employed, which could affect the observed outcome. In this regard, the use of single live-cell confocal imaging is especially useful since it allows cell tracking and detection of subtle changes in cell signalling. Intracellular signalling is often characterized by a heterogeneous response to an external stimulus in a cell population, making them difficult to detect in whole population studies using conventional biochemical methods^39^.

The effect of E2 on TLR5-dependent NF-κB signalling may be explained by either a decreased number of TLR5 receptors, as seen by the decreased expression of TLR5 after E2 supplementation, or through a direct interaction of ERs with the NF-κB transcription factors in the nucleus^40^. The inhibition of NF-κB activity suggests that E2 has an anti-inflammatory effect on TLR5 mediated immune response, as it has been previously described for TLR4^36^,^37^. However, we also observed that when incubated with E2, MCF7 cells increased TLR5-mediated AP-1 activation. The activation of the AP-1 transcription factor orchestrates the expression of many pro-inflammatory genes and further studies will be needed to decipher how E2 control inflammation^41^. But, how is this effect mediated? Modulation of NF-κB and AP-1 transcription factors by E2 is mediated by different ERs. As previously mentioned the nuclear ERs can interact directly with RelA and AP-1 transcription factors to modify their activity^40^,^42^. Previous studies have shown negative interactions between nuclear ERs and NF-κB activity. However, each nuclear ER can have a different effect on the transcription of target genes, with important differences reported between studies regarding the role of either ERα or ERβ in those interactions^37^,^43^. Our results suggest that the E2 effect on TLR5-mediated NF-κB transcription activity is directed through ERα and GPR30, while ERα and ERβ are responsible for the E2 effects on AP-1 activity. ERα plays a prominent role in the modulation of TLR5 signalling since it reproduces the observed E2 effect in both NF-κB and AP-1 activation. On the other hand, ERβ was able to modulate AP-1 activity but no effect on NF-κB was observed. This is in agreement with recent data produced by genome-wide mapping of ERβ binding sites in MCF7 cells, which suggested a functional association between ERβ and AP-1 signalling^44^. When we evaluated the role of GPR30 in TLR5 signalling we observed that it was able to decrease NF-κB activation but no effect was observed in AP-1 transcription activity. These results are surprising since GPR30 has been described to induce MAPK signalling^17^, suggesting that cross-talk between signalling pathways (in this case TLR5 and GPR30) has a great influence in the final outcome of GPR30 intracellular signalling.

In conclusion, we have shown for the first time the dynamics of NF-κB activation by TLR5 and how they are modulated by E2. TLR5 presents very similar dynamics to that previously described for TLR4. E2 is able to decrease TLR5 induced activation of NF-κB, while increasing AP-1 activation. This effect is coordinated through specific ERs and is dependent on the transcription factor and seems to occur in the nucleus since no changes in the dynamics of NF-κB translocation were observed. Understanding TLR5 signalling and how it is modulated can be of special importance for the treatment of a great number of pathologies that are influenced by both TLR5 and sex hormones.

## Methods

### Materials

Flagellin was supplied by Invivogen (Fla-ST Cat. Code tlrl-stfla). β-estradiol water-soluble was supplied by Sigma (Cat. No. E4389). Propylpyrazoletriol (PPT, Cat. No. 1426), dyarilpropiolnitrile (DPN, Cat. No. 1494), G1 (Cat. No. 3577), methylpiperidinopyrazole (MPP dihydrochloride, Cat. No. 1991), G15 (Cat. No. 3678) and pyrazolo [1,5-a] pyrimidin (PHTPP, Cat. No. 2662) were supplied by Tocris bioscience. X-tremeGENE HP DNA transfection reagent was supplied by Roche (Cat. No. 06366236001).

### Cell lines and culture

MCF-7 cells were cultured at 37 °C in an atmosphere with 5% CO_2_ in DMEM-F12 phenol free media (Gibco, Life technologies, Cat. No. 21041025, Paisley, UK) supplemented with 1% Penicillin and Streptomycin (P/S Sigma, Cat. No. PO781-100ML, St. Louis, MO), 10% charcoal stripped fetal bovine serum (FBS, Cat. No. DE14-820E, Lonza, UK), 1% L-glutamine (Sigma, Cat. No. G7513, Irvine, UK) and 160 ng/ml Insulin (Human recombinant insulin, Gibco Invitrogen, Cat. No. 12585-014, Denmark) unless otherwise stated.

### Prediction of transcription factor binding sites in the TLR5 promoter

A 3.5 kb TLR5 promoter region (from −2243 to +1280) was obtained from the Ensembl database (TLR5-001; transcript ID: ENST00000366881) and putative binding sequences for ERα were identified using PROMO 3.0 (http://alggen.lsi.upc.es/cgi-bin/promo_v3/promo/promoinit.cgi?dirDB=TF_8.3)^45^. Sequences were aligned using the free software SerialCloner 2.6.1.

### Reporter plasmids

We employed four different reporter plasmids: (i) a reporter vector that contains 5x κB binding sites driving the expression of secreted alkaline phosphatase (SEAP), used to measure NF-κB transcription activity (pNifty2-SEAP); (ii) a reporter vector that contains 5x TPA responsive elements (TRE) driving the expression of secreted alkaline phosphatase (SEAP). AP-1 binds to TRE and transcription activity is related to SEAP secretion (pNifty-3-A-SEAP); (iii) a vector expressing RelA fused to the Discosoma sp. red fluorescent protein dsRed-Express (RelA-dsRedxp), used to evaluate RelA translocation into the nucleus; and (iv) a reporter vector containing a 3.5 kb promoter region from the TLR5 locus driving SEAP expression (hTLR5p-SEAP) was used to evaluate TLR5 gene expression. This plasmid was generated by exchanging the 5xNF-κB binding sites and the ELAM promoter from the pNifty2-SEAP plasmid (Invitrogen) for a 3.5 kb TLR5 promoter region (from −2243 to +1280) (Fig. S1).

### Single live-cell fluorescent imaging

Confocal microscopy was carried out as described in^5^ with Zeiss LSM510 and LSM780 systems, using either 40x Fluar 1.4 NA or 63x Plan Apochromat 1.4 NA objectives. Treatment of cells with flagellin (100 ng/ml final concentration) after a 60 min pre-stimulation period was carried out *in-situ* between imaging acquisitions by replacing one tenth of the medium volume in the dish with the appropriate solution. Each experiment was carried out at least three times with at least 20 cells obtained per replicate. CellTracker^46^ was used for data extraction from time series images of cells. For p65-dsRedxp fusion proteins, mean fluorescence intensities were calculated for each time point for both nuclei and whole cell boundaries then nuclear:total fluorescence intensity ratios were determined.

### Reporter gene analysis

TLR5 expression, NF-κB and AP-1 transcription activity were evaluated using MCF7 cells grown in 12-well plates and transiently transfected with hTLR5p-SEAP, pNifty2-SEAP or pNifty3-A-SEAP (Invivogen) reporter plasmids, respectively. The optimised ratio of DNA:X-tremeGENE HP DNA was 1 μg DNA with 3 μl X-tremeGENE HP. Samples were pre-incubated for 24 h with the pertinent hormonal treatment and stimulated for 24 h with 100 ng/ml of flagellin unless otherwise stated. For each experiment, SEAP in the supernatant was detected using either QUANTI-Blue^TM^ (Invivogen) or NovaBright™ Secreted Placental Alkaline Phosphatase (SEAP) Enzyme Reporter Gene Chemiluminescent Detection System 2.0 kit (Invitrogen) following the manufacturer’s protocol. Samples assayed using the QUANTI-Blue^TM^ were quantified as OD at 620 nm using a microplate reader (Multiskan). When higher sensitivity was required in the assay, Novabright^TM^ kit was employed. The results from this chemiluminescent assay were read using a Sirius Luminometer (Berthold detection systems; Geneflow Staffs. UK). Data from all experiments are reported as the fold induction of SEAP activity over untreated controls.

### Western blotting

MCF7 cells were pre-incubated with or without 10 nM E2 for 24 h before the stimulation with 100 ng/ml of flagellin. Following treatment, cells were harvested at 0, 15, 30, 45, 60 and 90 min in 200 μl lysis buffer (40 mM Tris-Cl, pH 6.8, 1% w/v SDS, 1% v/v glycerol, 1% v/v β-mercaptoethanol, 0.01% w/v bromophenol blue). The lysates were boiled for 10 min and the proteins separated by polyacrylamide gel (12%) electrophoresis and transferred to nitrocellulose membranes. Membranes were probed using the following antibodies: anti-IκBα (#9242, Cell Signalling, MA, USA) and anti-alpha tubulin (#2144, Cell Signalling, MA, USA). Band intensity was quantified using a BioRad GS-710 Densitometer and Quantity One software (Version 4.5.0).

### Analysis of gene expression

To evaluate the effect of E2 on TLR5 gene expression, MCF7 cells were incubated with (10 nM) or without E2 for and collected after 1, 2, 4, 8 and 24 h. To determine the effect of E2 on TLR5-mediated IκBα expression, MCF7 cells where pre-incubated with (10 nM) or without E2 for 24 h. Then they were treated with 100 ng/ml of flagellin and collected at 0, 0.5, 2 and 6 h post-stimulation. To determine the effect of E2 on TLR5-mediated IL-1ra and ERα expression, MCF7 cells where pre-incubated with (10 nM) or without E2 for 24 h. Then they were treated with 100 ng/ml of flagellin and collected at 1, 2, 4 and 8 h post-stimulation. Gene expression was evaluated using quantitative real-time PCR amplification (qPCR). Primers are listed in Table S1. Amplified qPCR products were sequenced with forward and reverse primers to verify the resulting product. Total RNA from the samples was treated with DNase I (DNA-free kit; Ambion.) to remove genomic DNA contamination from samples. First-strand cDNA synthesis was performed using High Capacity cDNA Reverse Transcription Kit (AppiedBiosystems) according to manufacturer’s instructions. Quantitative PCR assays were performed using the SYBR Green Jump Start (Sigma) and Mx3005 P QPCR (Stratagene, Waldbronn, Germany). Quantitative PCR data were analysed using MxPro QPCR software version 4.01. and the comparative CT method. Β-actin and B2M genes were used as references genes for normalization of qPCR data.

### Statistical analysis

Data of NF-κB and AP-1 transcription activity are reported as the fold induction of SEAP activity over untreated controls. Unless otherwise stated, statistical analysis was performed using ANOVA (SPSS version 19.0; SPSS inc, Chicago, IL, USA). When ANOVA revealed a significant effect, values were compared using the Bonferroni test and were considered significant at p < 0.05. For imaging data, the average and standard deviation of the nuclear:total ratio from the 60 min pre-stimulation period were calculated and a threshold set which was four standard deviations above pre-stimulation average. An NF-κB response was characterised as being a transient rise in nuclear:total ratio with more than three points above this threshold in the first 6 h following treatment. For responding cells, amplitude and time until first peak were compared using a Mann-Whitney test.

## Additional Information

**How to cite this article**: Caballero, I. *et al*. Understanding the dynamics of Toll-like Receptor 5 response to flagellin and its regulation by estradiol. *Sci. Rep.* **7**, 40981; doi: 10.1038/srep40981 (2017).

**Publisher's note:** Springer Nature remains neutral with regard to jurisdictional claims in published maps and institutional affiliations.

## Supplementary Material

Supplementary Data

## References

[b1] Takeuchi O. & Akira S. Pattern recognition receptors and inflammation. Cell 140, 805–820, doi: 10.1016/j.cell.2010.01.022 (2010). 20303872

[b2] Hartl D. . Innate immunity in cystic fibrosis lung disease. Journal of cystic fibrosis: official journal of the European Cystic Fibrosis Society 11, 363–382, doi: 10.1016/j.jcf.2012.07.003 (2012). 22917571

[b3] Papadimitraki E. D., Bertsias G. K. & Boumpas D. T. Toll like receptors and autoimmunity: a critical appraisal. Journal of autoimmunity 29, 310–318, doi: 10.1016/j.jaut.2007.09.001 (2007). 17959357

[b4] Rakoff-Nahoum S. & Medzhitov R. Toll-like receptors and cancer. Nature reviews. Cancer 9, 57–63, doi: 10.1038/nrc2541 (2009). 19052556

[b5] Ashall L. . Pulsatile stimulation determines timing and specificity of NF-kappaB-dependent transcription. Science 324, 242–246, doi: 10.1126/science.1164860 (2009). 19359585 PMC2785900

[b6] Drexler S. K. & Foxwell B. M. The role of toll-like receptors in chronic inflammation. The international journal of biochemistry & cell biology 42, 506–518, doi: 10.1016/j.biocel.2009.10.009 (2010). 19837184

[b7] Wang Y. . Interactions among oscillatory pathways in NF-kappa B signaling. BMC systems biology 5, 23, doi: 10.1186/1752-0509-5-23 (2011). 21291535 PMC3050740

[b8] Bakstad D., Adamson A., Spiller D. G. & White M. R. Quantitative measurement of single cell dynamics. Current opinion in biotechnology 23, 103–109, doi: 10.1016/j.copbio.2011.11.007 (2012). 22137453

[b9] Spiller D. G., Wood C. D., Rand D. A. & White M. R. Measurement of single-cell dynamics. Nature 465, 736–745, doi: 10.1038/nature09232 (2010). 20535203

[b10] Zambrano S., De Toma I., Piffer A., Bianchi M. E. & Agresti A. NF-kappaB oscillations translate into functionally related patterns of gene expression. Elife 5, doi: 10.7554/eLife.09100 (2016). PMC4798970 26765569

[b11] Levine J. H., Lin Y. & Elowitz M. B. Functional roles of pulsing in genetic circuits. Science 342, 1193–1200, doi: 10.1126/science.1239999 (2013). 24311681 PMC4100686

[b12] Lee T. K. . A noisy paracrine signal determines the cellular NF-kappaB response to lipopolysaccharide. Science signaling 2, ra65, doi: 10.1126/scisignal.2000599 (2009). 19843957 PMC2778577

[b13] Reardon S. Infections reveal inequality between the sexes. Nature 534, 447, doi: 10.1038/534447a (2016). 27337319

[b14] Calippe B. . 17Beta-estradiol promotes TLR4-triggered proinflammatory mediator production through direct estrogen receptor alpha signaling in macrophages *in vivo*. Journal of immunology 185, 1169–1176, doi: 10.4049/jimmunol.0902383 (2010). 20554954

[b15] Straub R. H. The complex role of estrogens in inflammation. Endocrine reviews 28, 521–574, doi: 10.1210/er.2007-0001 (2007). 17640948

[b16] Bjornstrom L. & Sjoberg M. Mechanisms of estrogen receptor signaling: convergence of genomic and nongenomic actions on target genes. Molecular endocrinology 19, 833–842, doi: 10.1210/me.2004-0486 (2005). 15695368

[b17] Maggiolini M. & Picard D. The unfolding stories of GPR30, a new membrane-bound estrogen receptor. The Journal of endocrinology 204, 105–114, doi: 10.1677/JOE-09-0242 (2010). 19767412

[b18] Kassi E. & Moutsatsou P. Estrogen receptor signaling and its relationship to cytokines in systemic lupus erythematosus. Journal of biomedicine & biotechnology 2010, 317452, doi: 10.1155/2010/317452 (2010). 20617147 PMC2896666

[b19] Zarember K. A. & Godowski P. J. Tissue expression of human Toll-like receptors and differential regulation of Toll-like receptor mRNAs in leukocytes in response to microbes, their products, and cytokines. Journal of immunology 168, 554–561 (2002). 10.4049/jimmunol.168.2.554 11777946

[b20] Caballero I. . Human trophoblast cells modulate endometrial cells nuclear factor kappaB response to flagellin *in vitro*. PloS one 8, e39441, doi: 10.1371/journal.pone.0039441 (2013). 23320062 PMC3540055

[b21] Cai Z. . Activation of Toll-like receptor 5 on breast cancer cells by flagellin suppresses cell proliferation and tumor growth. Cancer research 71, 2466–2475, doi: 10.1158/0008-5472.CAN-10-1993 (2011). 21427357 PMC3074302

[b22] Carvalho F. A., Aitken J. D., Vijay-Kumar M. & Gewirtz A. T. Toll-like receptor-gut microbiota interactions: perturb at your own risk! Annu Rev Physiol 74, 177–198, doi: 10.1146/annurev-physiol-020911-153330 (2012). 22035346

[b23] Munoz N. . Mucosal administration of flagellin protects mice from Streptococcus pneumoniae lung infection. Infect Immun 78, 4226–4233, doi: 10.1128/IAI.00224-10 (2010). 20643849 PMC2950348

[b24] Munukka E. . Adipocytes as a Link Between Gut Microbiota-Derived Flagellin and Hepatocyte Fat Accumulation. PloS one 11, e0152786, doi: 10.1371/journal.pone.0152786 (2016). 27035341 PMC4817958

[b25] Lion M. . Interaction between p53 and estradiol pathways in transcriptional responses to chemotherapeutics. Cell Cycle 12, 1211–1224, doi: 10.4161/cc.24309 (2013). 23518503 PMC3674086

[b26] Foust-Wright C. E. . Hormone Modulation of Toll-Like Receptor 5 in Cultured Human Bladder Epithelial Cells. Reprod Sci, doi: 10.1177/1933719116667489 (2016). 27651177

[b27] Price J. C., Bromfield J. J. & Sheldon I. M. Pathogen-associated molecular patterns initiate inflammation and perturb the endocrine function of bovine granulosa cells from ovarian dominant follicles via TLR2 and TLR4 pathways. Endocrinology 154, 3377–3386, doi: 10.1210/en.2013-1102 (2013). 23825132

[b28] Price J. C. & Sheldon I. M. Granulosa cells from emerged antral follicles of the bovine ovary initiate inflammation in response to bacterial pathogen-associated molecular patterns via Toll-like receptor pathways. Biol Reprod 89, 119, doi: 10.1095/biolreprod.113.110965 (2013). 24089202

[b29] Lee A. V., Oesterreich S. & Davidson N. E. MCF-7 cells–changing the course of breast cancer research and care for 45 years. J Natl Cancer Inst 107, doi: 10.1093/jnci/djv073 (2015). 25828948

[b30] Smith M. F. Jr., Eidlen D., Arend W. P. & Gutierrez-Hartmann A. LPS-induced expression of the human IL-1 receptor antagonist gene is controlled by multiple interacting promoter elements. Journal of immunology 153, 3584–3593 (1994). 7930581

[b31] Junkin M. . High-Content Quantification of Single-Cell Immune Dynamics. Cell Rep 15, 411–422, doi: 10.1016/j.celrep.2016.03.033 (2016). 27050527 PMC4835544

[b32] Turner D. A. . Physiological levels of TNFalpha stimulation induce stochastic dynamics of NF-kappaB responses in single living cells. Journal of cell science 123, 2834–2843, doi: 10.1242/jcs.069641 (2010). 20663918 PMC2915884

[b33] Zeitlin P. L. Cystic fibrosis and estrogens: a perfect storm. The Journal of clinical investigation 118, 3841–3844, doi: 10.1172/JCI37778 (2008). 19033654 PMC2582937

[b34] Galien R. & Garcia T. Estrogen receptor impairs interleukin-6 expression by preventing protein binding on the NF-kappaB site. Nucleic acids research 25, 2424–2429 (1997). 9171095 10.1093/nar/25.12.2424 PMC146754

[b35] Sun W. H., Keller E. T., Stebler B. S. & Ershler W. B. Estrogen inhibits phorbol ester-induced I kappa B alpha transcription and protein degradation. Biochem Biophys Res Commun 244, 691–695, doi: 10.1006/bbrc.1998.8324 (1998). 9535726

[b36] Dodel R. C., Du Y., Bales K. R., Gao F. & Paul S. M. Sodium salicylate and 17beta-estradiol attenuate nuclear transcription factor NF-kappaB translocation in cultured rat astroglial cultures following exposure to amyloid A beta(1–40) and lipopolysaccharides. J Neurochem 73, 1453–1460 (1999). 10501189 10.1046/j.1471-4159.1999.0731453.x

[b37] Ghisletti S., Meda C., Maggi A. & Vegeto E. 17beta-estradiol inhibits inflammatory gene expression by controlling NF-kappaB intracellular localization. Molecular and cellular biology 25, 2957–2968, doi: 10.1128/MCB.25.8.2957-2968.2005 (2005). 15798185 PMC1069609

[b38] Xing D. . Estrogen modulates NFkappaB signaling by enhancing IkappaBalpha levels and blocking p65 binding at the promoters of inflammatory genes via estrogen receptor-beta. PloS one 7, e36890, doi: 10.1371/journal.pone.0036890 (2012). 22723832 PMC3378567

[b39] Mullassery D., Horton C. A., Wood C. D. & White M. R. Single live-cell imaging for systems biology. Essays in biochemistry 45, 121–133, doi: 10.1042/BSE0450121 (2008). 18793128 PMC2796723

[b40] Quaedackers M. E., van den Brink C. E., van der Saag P. T. & Tertoolen L. G. Direct interaction between estrogen receptor alpha and NF-kappaB in the nucleus of living cells. Molecular and cellular endocrinology 273, 42–50, doi: 10.1016/j.mce.2007.05.002 (2007). 17590503

[b41] Shan Z. X. . Transcription factor Ap-1 mediates proangiogenic MIF expression in human endothelial cells exposed to Angiotensin II. Cytokine 53, 35–41, doi: 10.1016/j.cyto.2010.09.009 (2011). 21030269

[b42] Dahlman-Wright K. . Interplay between AP-1 and estrogen receptor alpha in regulating gene expression and proliferation networks in breast cancer cells. Carcinogenesis 33, 1684–1691, doi: 10.1093/carcin/bgs223 (2012). 22791811

[b43] Paech K. . Differential ligand activation of estrogen receptors ERalpha and ERbeta at AP1 sites. Science 277, 1508–1510 (1997). 9278514 10.1126/science.277.5331.1508

[b44] Zhao C. . Genome-wide mapping of estrogen receptor-beta-binding regions reveals extensive cross-talk with transcription factor activator protein-1. Cancer research 70, 5174–5183, doi: 10.1158/0008-5472.CAN-09-4407 (2010). 20501845

[b45] Farre D. . Identification of patterns in biological sequences at the ALGGEN server: PROMO and MALGEN. Nucleic acids research 31, 3651–3653 (2003). 12824386 10.1093/nar/gkg605 PMC169011

[b46] Du C. J., Marcello M., Spiller D. G., White M. R. & Bretschneider T. Interactive segmentation of clustered cells via geodesic commute distance and constrained density weighted Nystrom method. Cytometry. Part A: the journal of the International Society for Analytical Cytology 77, 1137–1147, doi: 10.1002/cyto.a.20993 (2010). 21069796

