# Supplementary material for: Understanding the dynamics of Toll-like Receptor 5 response to flagellin and its regulation by estradiol
Source: Sci Rep. 2017 Jan 23;7:40981. doi: 10.1038/srep40981 (PMC5256028; doi:10.1038/srep40981)
Supplement: Supplementary Data [file srep40981-s1.pdf]

Understanding the dynamics of Toll-like Receptor 5 response to flagellin and its regulation by estradiol

Ignacio Caballero[2,3], James Boyd[4], Carmen Almiñana[2], Javier A. Sánchez-López[2], Shaghayegh Basatvat[2], Mehrnaz Montazeri[2], Nasim Maslehat Lay[2], Sarah Elliott[2], David G. Spiller[4], Michael R. White[4], Alireza Fazeli[1,2,]*

[1]Institute of Biomedicine and Translational Medicine, Department of Pathophysiology, University of Tartu, Tartu, Estonia

[2]Academic Unit of Reproductive and Developmental Medicine, Department of Oncology and Metabolism, The Medical School, University of Sheffield, Sheffield, United Kingdom.

[3]ISP, INRA, Université Tours, 37380, Nouzilly, France.

[4]Michael Smith Building, Faculty of Life Sciences, University of Manchester, Manchester, United Kingdom.

*Fazeli@ut.ee

Supplementary data

Table S1. Primers used for evaluation of gene expression by quantitative real-time PCR amplification.

Gene	Forward primer	Reverse primer	Annealing T (°C)	Product size (bp)
TLR5	CACCAAACCAGGGATGCTAT	CCTGTGTATTGATGGGCAAA	58	111
IκBα	CCCTACACCTTGCCTGTGAG	CGTGTGGCCATTGTAGTTGG	62	116
IL-1ra	CCAGCAAGATGCAAGCCTTCAGAAT	CCAGACTTGACACAGGACAGGC	60	129
ESR1	GAATCTGCCAAGGAGACTCG	ATCTCTCTGGCGCTTGTGTT	60	288
B-actin	CAAGATCATTGCTCCTCCTG	ATCCACATCTGCTGGAAGG	62	152
B2M	TATGCCTGCCGTGTGAACCA	GCGGCATCTTCAAACCTCCA	62	98

Figure S1. Schematic depiction of the three different reporter constructs used: i) a reporter vector that contains 5x κB binding sites and the ELAM minimum promoter driving the expression of secreted alkaline phosphatase (SEAP), used to measure NF-κB transcription activity (pNifty2-SEAP); ii) a reporter vector that contains 5x TPA responsive elements (TRE) and a the IFN-β minimum promoter driving the expression of secreted alkaline phosphatase (SEAP). AP-1 binds to TRE and transcription activity is related to SEAP secretion (pNifty-3-A-SEAP); iii) a vector expressing RelA fused to the Discosoma sp. red fluorescent protein dsRed-Express (RelA-dsRedxp), used to evaluate RelA translocation pulses into the nucleus; and iv) a reporter vector containing a 3.5 kb promoter region from the TLR5 locus driving SEAP expression, used to evaluate TLR5 gene expression.

Figure S2. Analysis of RelA-dsRedxp dynamics in MCF7 cells responding or not to flagellin stimulation. MCF7 cells were transiently transfected with RelA-dsRedxp and pre-incubated or not (control) with 10 nM E2 for 24 h before stimulation with 100 ng/ml of flagellin. (A and B) Time course of nuclear:total fluorescence intensities of RelA-dsRedxp in control MCF7 cells responding (A) or not (B) to flagellin stimulation. (C and D) Time course of nuclear:total fluorescence intensities of RelA-dsRedxp in MCF7 cells pre-incubated with 10 nm E2 responding (C) or not (D) to flagellin stimulation.

Figure S3. Estradiol modulates ESR1 gene expression. Real-time qPCR analysis of ESR1 gene expression in MCF7 cells cultured in the presence of 10 nM E2 or not (control) and stimulated with 100 ng/ml of flagellin for 1, 2, 4 and 8 h before collection. Data are

representative of at least five three independent experiments. Error bars denote SEM. The asterix (*) indicates a significant difference ($p<0.05$).

Figure S4. Estrogen receptors modulate TNF-α activation of NF-κB transcription activity. MCF7 cells transiently transfected with pNifty2-SEAP were pre-incubated with ER agonists PPT, DPN or G1 (1000 nM) for 24 h and then stimulated with 100 ng/ml of flagellin and supernatant collected at 6 and 24 h. Samples were analyzed using NovaBrightTM Secreted Placental Alkaline Phosphatase (SEAP) Enzyme Reporter Gene Chemiluminescent Detection System 2.0. Control Data of NF-kB activity are reported as the fold induction of SEAP activity over untreated controls. Different letters (a and b) indicate significant differences ($p<0.05$).

Figure S1

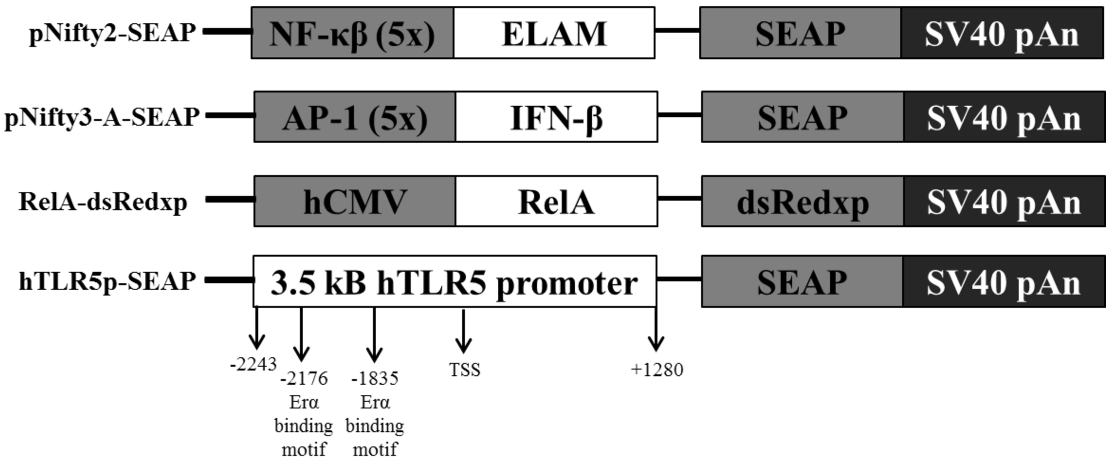
pNifty2-SEAP
NF-κβ (5x)
ELAM
SEAP
SV40 pAn
pNifty3-A-SEAP
AP-1 (5x)
IFN-β
SEAP
SV40 pAn
RelA-dsRedxp
hCMV
RelA
dsRedxp
SV40 pAn
hTLR5p-SEAP
3.5 kB hTLR5 promoter
SEAP
SV40 pAn
-2243
-2176 Erα binding motif
-1835 Erα binding motif
TSS
+1280

Figure S2

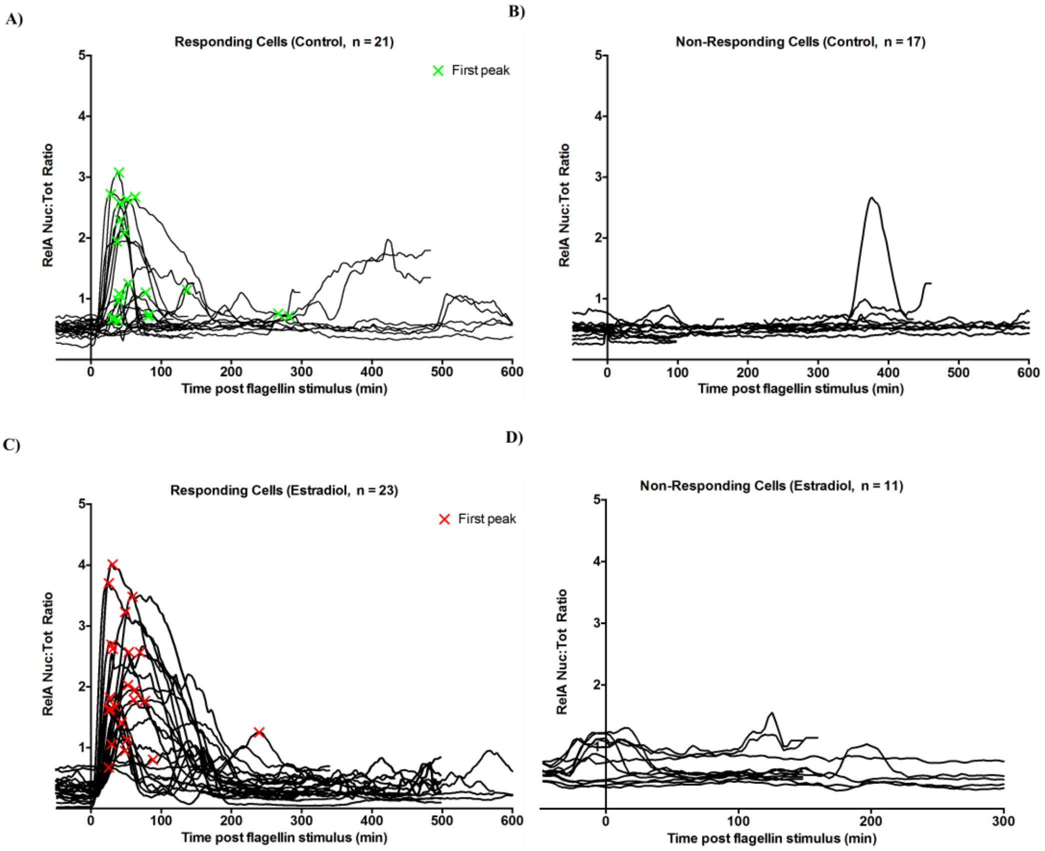

A)
Responding Cells (Control, n = 21)
First peak
RelA Nuc:Tot Ratio
Time post flagellin stimulus (min)
B)
Non-Responding Cells (Control, n = 17)
RelA Nuc:Tot Ratio
Time post flagellin stimulus (min)
C)
Responding Cells (Estradiol, n = 23)
First peak
RelA Nuc:Tot Ratio
Time post flagellin stimulus (min)
D)
Non-Responding Cells (Estradiol, n = 11)
RelA Nuc:Tot Ratio
Time post flagellin stimulus (min)

Figure S3

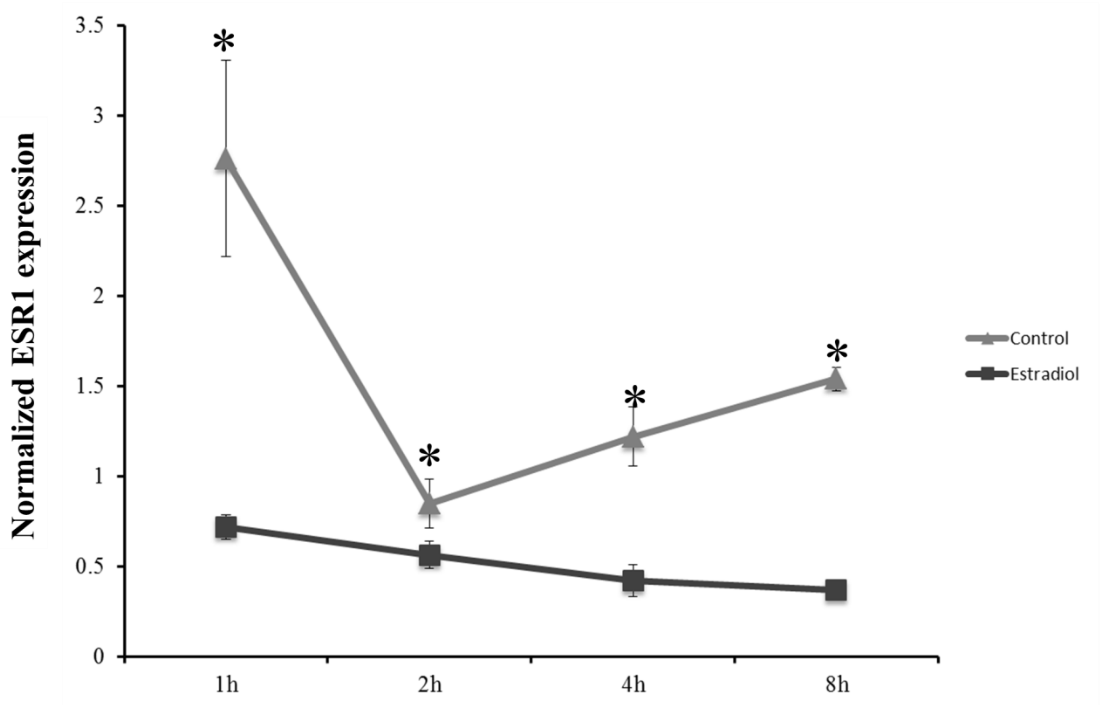
Normalized ESR1 expression
3.5
3
2.5
2
1.5
1
0.5
0
1h
2h
4h
8h
*
*
*
*
Control
Estradiol

Figure S4

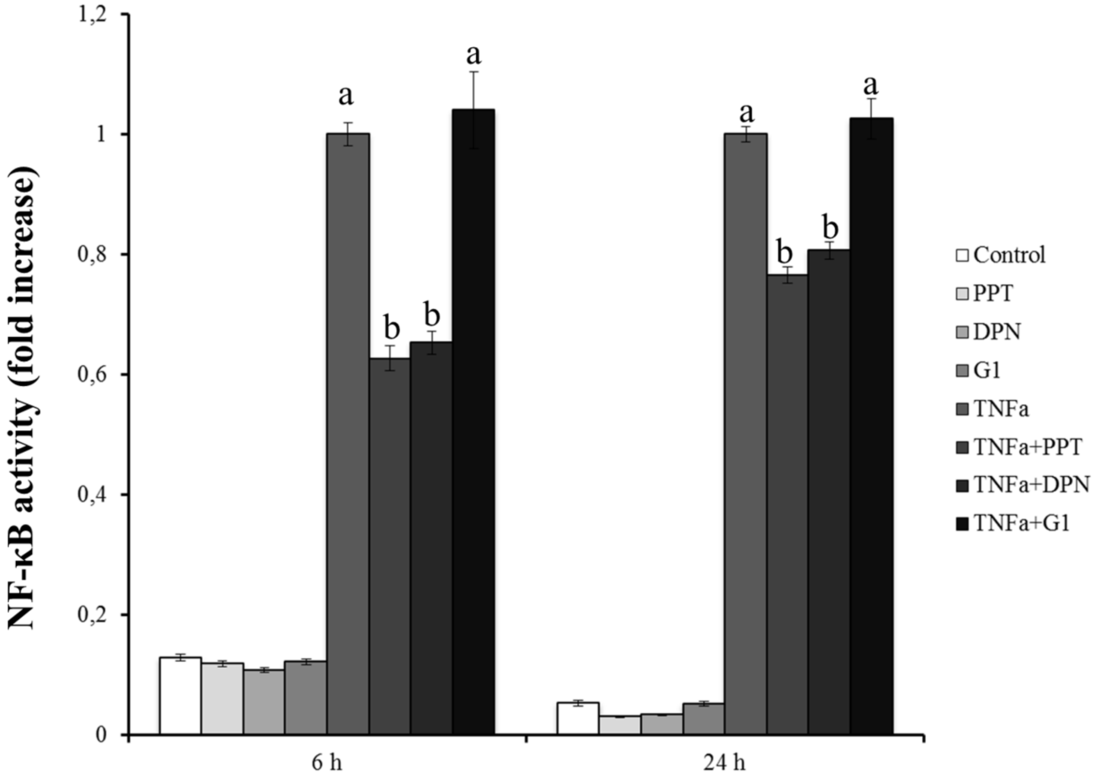

NF-κB activity (fold increase)
1,2
1
0,8
0,6
0,4
0,2
0
a
b
b
a
a
b
b
a
6 h
24 h
Control
PPT
DPN
G1
TNFa
TNFa+PPT
TNFa+DPN
TNFa+G1
